# Retrieving a file binder from a bookshelf using pseudo-curved trajectory generation for a foldable robotic hand

**DOI:** 10.1038/s41598-024-62699-4

**Published:** 2024-05-22

**Authors:** Hidetoshi Ikeda, Takumi Saeki, Kota Takabayashi

**Affiliations:** 1 Department of Engineering, Niigata Institute of Technology, 1719 Fujihashi, Kashiwazaki, 945-1195 Niigata Japan; 2 Department, National Institute of Technology, Toyama college, 13 Hongouchou, Toyama, 939-8630 Toyama Japan

**Keywords:** Robotic hand, Foldable hand, Retrieving file binder, Object tilting, Bookshelf, Pseudo-curved trajectory, Mechanical engineering, Electrical and electronic engineering

## Abstract

Human-assistive robots need to perform trajectory making for and control of a robotic hand along the many rotating mechanisms in our living spaces. If such trajectory control can be performed without high-cost sensors, certainly a significant cost reduction in building the robot will be achieved. This paper describes a method of retrieving a file binder by generating a pseudo-curved trajectory for tilting it using a simple system. A simple claw mechanism with a switch sensor to grasp an object was designed and 3D-printed, and it was attached to a 6-DOF foldable robotic hand developed by the authors. A method for generating a pseudo-curved trajectory using the switch sensor was developed, and the robotic hand was successfully moved along this trajectory to tilt and grasp a file binder to retrieve it from a bookshelf. Experiments to clarify the success rate were also conducted, and it was found that the results depend on the rotational speed of manipulator links and the vibration of the claw mechanism link. A rubber sponge was added to give flexibility to the claw mechanism, which significantly improved the success rate. Furthermore, a control system to recover from tilting failure was constructed, and its effectiveness was validated by experiments.

## Introduction

Many multi-finger hand mechanisms have been widely researched: a robotic hand with two fingers and two axes^1^, a robotic hand with two fingers and five axes^2^, a robotic hand with four fingers^3^, and a human-like robotic hand with five fingers^4,5^. In addition, human-like robotic hands have been studied for application as robotic prosthetic hands^6–8^. A multi-finger hand has the potential to accomplish advanced tasks; however, such hands have high structural complexity and are difficult to control. Therefore, it is desirable to reduce the numbers of fingers and axes in the robotic hand while retaining the ability to handle and control various objects.

Numerous underactuated robotic hands or grippers that have few actuators for driving joints have been studied^9–13^. Research has also been conducted on how to allocate actuators to the degrees of freedom (DOFs) of robotic hands^14^.

The inside of a finger is relatively narrow, and robotic hands with a tendon drive mechanism have been extensively researched^15^. Other approaches to tendon drive mechanisms are an integrated linkage-spring-tendon compliant anthropomorphic robotic hand^16^, a tendon-driven robotic hand with underactuated robotic fingers that can open the cap of a glass bottle and pour coffee^17^, and a robotic hand with a tendon drive mechanism and torsional springs that can adapt to the form of an object and grasp it^18^.

Soft robotic hands that can handle delicate objects have also been investigated: a robotic hand with four soft fingers for handling groceries^19^, a robotic hand with three soft fingers that can sort various types of fruit^20^, and soft hands or grippers for handling delicate marine organisms^21,22^. In addition, robotic hands with soft fingers that are arranged similar to those of a human hand have been studied and used to sort ripe tomatoes^23^. A prosthetic soft robotic arm was also developed to grasp various objects^24^.

A robotic hand that uses the jamming of a granular material instead of fingers has been studied^25^. A robotic hand that uses a jamming transition and a tendon drive mechanism has also been studied^26^. A gripper mechanism with many pins that can conform to the shape of an object has been developed^27^.

Multi-finger hands with additional functions that human hands lack have been studied. Research has been conducted on a hand that has two fingers and a sliding mechanism for precise handling using the surface of the fingers^28^, a hand with a mechanism that allows four fingers to fully rotate^29^, and a hand with a ball mechanism at the tip of each finger^30^.

A great deal of effort has been made on the development of robotic hands other than those described above, and a review of robotic hands was reported by Bicchi^31^. Humans bend their fingers and change the shape of their hands to perform various tasks, and many studies have also reported on the taxonomy of the grasping mechanism of human hands and have evaluated its capability^32–36^.

The present authors previously designed a robotic hand that has a foldable mechanism, a wide movable range of joints, and the ability to use the backs and sides of its fingers^37^. The hand has 6 DOFs and can perform various tasks, such as placing, grasping, pinching, pulling, and picking up an object and turning the pages of a book. The authors also previously presented a strategy for pinching and grasping a cylindrical object^38^.

In the present study, the authors developed a method for tilting a file binder and retrieving it from a bookshelf using the robotic hand.

### Related research on handling books, notebooks, and file binders using a human-assistive robot

Although research on using an assistive robot to retrieve a file binder from a bookshelf, which is the focus of this paper, has not yet been conducted, retrieval of a ring notebook from a bookshelf has been shown^39^, and several studies have been conducted on handling books. Kim et al.^40^ proposed a robotic library system using a mobile manipulator. Some research has studied the retrieval of a book from a bookshelf using a gripper with two thin fingers that are inserted parallel to the gap between books to grip a single book^41–44^.

In contrast, Prats et al. and Morales et al. developed a method of retrieving a book after tilting it with a robotic hand having three fingers controlled by impedance^45,46^. Bdiwi and Suchý studied a method for retrieving a book after tilting it using a gripper equipped with a torque sensor^47^. To perform the book tilting motion, it is necessary to move the robotic hand while following the nonlinear trajectory of the object as it tilts, and it is effective to use a system with a torque sensor for control, as described above.

However, high-performance torque sensors are expensive. Our living spaces contain many rotating systems, and a human-assistive robot has to move with the same trajectory when the robot handles objects with a rotational motion. A significant cost reduction to build the human-assistive robot can be achieved if the operation can be realized with a low-cost sensor.

This report describes a method for tilting and retrieving a file binder placed on a bookshelf using an inexpensive switch sensor that helps to generate a pseudo-nonlinear trajectory of the object during tilting. The proposed method is applicable to the control of various rotating devices and furniture in human living spaces and has the potential to revolutionize sensor configurations for human-assistive robots in a way that significantly reduces the required cost.

## Results

### Mechanism of the robot

**Figure 1:**
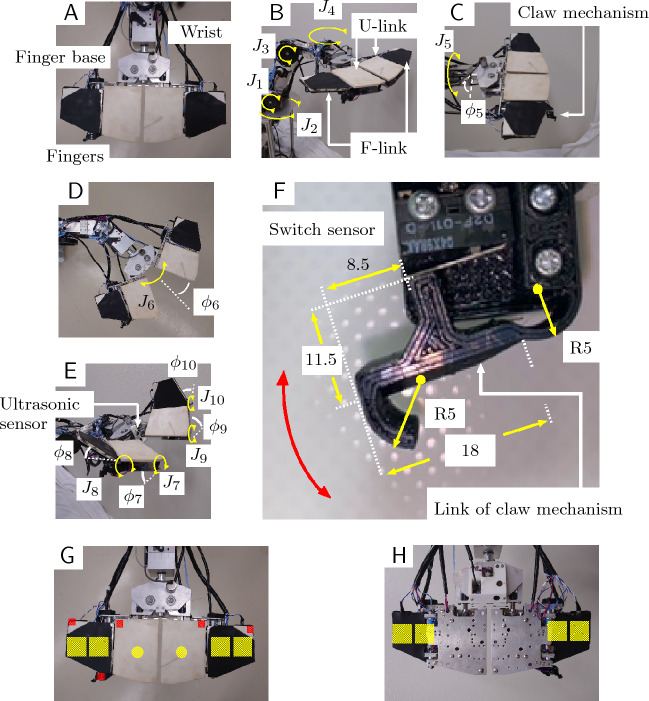
Details of the robotic hand mechanism. (**A**) Three parts of the hand: fingers, finger base, and wrist. (**B**) Axes of the manipulators. The shoulder axes are $J_1$ and $J_2$, and the elbow axes are $J_3$ and $J_4$. (**C**) Wrist axis rotating assembly, $J_5$, consisting of the finger base and fingers. One finger has a claw mechanism. (**D**) Axis set on the base controlling the angle of the fingers. The fingers are connected by multiple joints, which are driven by a single motor, $\phi _6$. (**E**) Axes that control the finger angle: $J_7$ to $J_{10}$. An ultrasonic sensor is installed on the base. (**F**) Approximate size of the claw mechanism. The mechanism was created with a 3D printer and is equipped with a touch sensor. (**G**) Positions of the switch sensor (small red squares) and the force (film) sensors on the front side of the finger (yellow circles and squares).(**H**) Positions of force (film) sensors on the back side of a finger (squares).

The robotic hand, manipulator links, and wheeled mechanism were developed in our laboratory (see "Methods" Section, Fig. 6E). The wheeled mechanism has two pairs of wheels, each of which consists of a left wheel and a right wheel. The front pair is casters, and the rear pair is driving wheels that have individually opposing drive systems. Two motors (Tsukasa Electric Co., Ltd., TG-85E-SU-47.9-KA, 24V) and two encoders (Autonics, E30S4-100-3-N-5) are arranged in the driving system.

**Table 1:** Specifications of the robot.

Overall length of wheeled mechanism	450 mm
Overall height of wheeled mechanism	270 mm
Radius of front wheels	15 mm
Radius of rear wheels	90 mm
Wheelbase	340 mm
Upper arm link of manipulator ($l_{uL}$)	300 mm
Forearm link of manipulator (length from elbow joint to the claw mechanism, $l_{fL}$)	570 mm
Thickness of wrist ($t_W$)	78 mm
Width of robot base ($b_B$)	84 mm
Thickness of robot base ($t_B$)	86 mm
Length of U-link ($l_U$)	132 mm
Length of F-link ($l_F$)	104 mm
Thickness of fingers ($t_F$)	30 mm
Width of fingers ($b_F$)	154 mm
Motion range of $\phi _1$	$-10$ deg $\le \phi _1 \le 95$ deg
Motion range of $\phi _2$	$-180$ deg $\le \phi _2 \le 180$ deg
Motion range of $\phi _3$	$-100$ deg $\le \phi _3 \le 100$ deg
Motion range of $\phi _4$	$-100$ deg $\le \phi _4 \le 100$ deg
Motion range of $\phi _5$	$-180$ deg $\le \phi _5 \le 180$ deg
Motion range of $\phi _6$	$-10$ deg $\le \phi _6 \le 50$ deg
Motion range of $\phi _7$	$-90$ deg $\le \phi _7 \le 90$ deg
Motion range of $\phi _8$	$-180$ deg $\le \phi _8 \le 60$ deg
Motion range of $\phi _9$	$-90$ deg $\le \phi _9 \le 90$ deg
Motion range of $\phi _{10}$	$-180$ deg $\le \phi _8 \le 60$ deg

**Figure 2:**
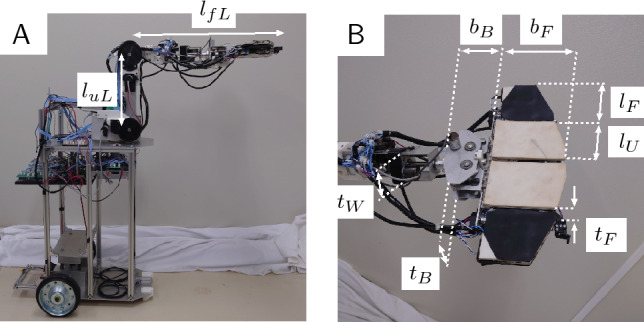
Notation for the manipulator and hand mechanism size parameters. (**A**) Manipulator links. (**B**) Hand mechanism components.

The robotic hand consists of three parts, namely the fingers, finger base, and wrist (Fig. 1A). The hand has right and left fingers, each of which has two links, U-link and F-link (Fig. 1B). The robotic hand is attached to the forearm link. The manipulator has shoulder axes ($J_1$ and $J_2$) and elbow axes ($J_3$ and $J_4$), with angles $\phi _i$, $i=1$–4, respectively (Fig. 6E). The motors and encoders are installed on each axis (for $J_1$ the $J_3$, the motor is Tsukasa Electric Co., Ltd., TG-85E-KU-113-KA, 24V, and the encoder is Nidec Copal Electronics, RE12D-100-201-1; for $J_4$, the motor is TG-101C-GU-581-KA, 24V, and the encoder is RECW20D-25-201-1). The wrist has an axis, $J_5$, that has a motor and an encoder (motor: Tsukasa Electric Co., Ltd., TG-101C-GU-581-KA, 24V; encoder: Nidec Copal Electronics, RE30E-360-213-1). The wrist is able to rotate the assembly that consists of the finger base and fingers (Fig. 1C) in the range $0 \le \phi _5 \le 180$ deg.

The robotic hand can transform its shape by folding or opening the fingers to conform to an object. Both fingers are attached to the finger base (Fig. 1D). The base has a motor and an encoder ($J_6$, Fig. 1D; motor: Tsukasa Electric Co., Ltd., TG-101C-GU-581-KA, 24V; encoder: Nidec Copal Electronics, RE12A-100-100-1). The base has two joints, and the joints are driven by the motor. It can change the finger angle, $\phi _6$, which has a movable range of $-10$ deg $\le \phi _6 \le 50$ deg (when fingers are vertical).

The U-links have motors and encoders to control the fingers ($J_7$-$J_{10}$, Fig. 1E); motor: Tsukasa Electric Co., Ltd., TG-85E-KU-113-KA, 24V; encoder: Nidec Copal Electronics, RE12D-100-201-1). Sponge rubber parts (Misumi Corp., PRGCW5, white) and rubber parts (2 mm thick, black) are glued on the front and back sides of the F-links. The joint angles of the left and right U-links ($\phi _7$ and $\phi _9$) are $-90$ deg $\le \phi _7 \le 90$ deg and $-90$ deg $\phi \le \phi _9 \le 90$ deg, respectively, and those of the F-links ($\phi _8$ and $\phi _{10}$) are -180 deg $\le \phi _8 \le 60$ deg and $-180$ deg $\le \phi _{10} \le 60$ deg, respectively (Fig. 1E). The motion ranges of the manipulator axes are shown in Table 1.

The claw mechanism to snag an object is installed on the side of the hand (Fig. 1C and F). The approximate size of the claw mechanism is shown in the Methods section, Fig. 7. The mechanism was made using a 3D printer. Part of the link is turned by the force when the link touches an object, and a microswitch (Omron Corp., D2MQ-01L-D, D2MQ-4L-1) is installed on the claw mechanism (Fig. 1C) to detect contact when the claw touches an object (the stroke of the claw mechanism is 4 mm). In this study, the claw mechanism was used for tilting the file binder as a first step in removing it from the bookshelf.

Two force sensors (film sensors) (Tekscan Corp., A201, High 445 N, 0-100 lb) are installed on the inside of the U-links of the fingers (positions shown by yellow circles in Fig. 1G). Five microswitch sensors (Omron Corp., D2F-01L-D) are installed on the inside of the U-links and claw mechanism (positions shown by red circles in Fig. 1G). When the hand makes contact with an object, the force sensors detect the contact. Four force sensors (film sensors) (Interlink Electronics Inc., FSR406) are installed on the front of each F-link (positions showed by yellow squares in Fig. 1G). Each sensor is covered by rubber. Four sensors are also installed on the back of each F-link (positions shown by yellow squares in Fig. 1H). The arrangement of switch sensors is shown in Fig. 1G. An ultrasonic sensor is installed on the base (Fig. 1E).

The motors and sensors were inexpensive, easily available, and selected to achieve the design target of handling a maximum object weight of 500 g.

The sizes of the manipulator and hand mechanism are shown in Fig. 2, and Table 1 shows the specifications of the robot.

#### Size and specifications of the manipulator and robotic hand

The notation for the size parameters of the manipulator and hand mechanism are shown in Fig. 2. Table 1 shows the specifications of the robot.

### System configuration

**Figure 3:**
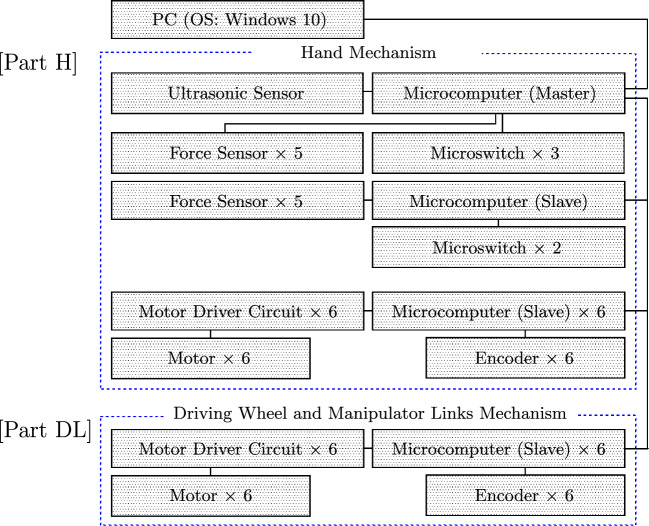
Configuration of the robot control system.

Figure 3 shows a configuration diagram for the system controls. As shown, the control system has three components: Part H, Part DL, and a PC. Part H is the control system for the hand mechanism and Part DL is the control system for the driving system (driving wheels and manipulator forearm and upper links). Part H includes a master microcomputer, which is connected to the PC.

[Part DL] The motors and encoders of the links of the manipulator (4 DOFs) and the driving wheel system (2 DOFs) are connected to each of the 6 motor driver circuits separately (Cytron Co., Ltd., MD10C). The motor driver circuits are connected to each microcomputer (Arduino Holdings, Arduino Leonardo). The motors and encoders of the robotic hand (6 DOFs) are similarly connected to each of the 6 motor driver circuits separately in Part A. In total, 6 microcomputers in Part A are used as slave computers.

[Part H] Two microswitches and force (film) sensors on the robotic hand (Fig. 1F−H) are connected to other microcomputers (Arduino Leonardo) in Part H.

In total, 6 microcomputers in Part H are used as slave computers. Five force (film) sensors, three microswitches, and the ultrasonic sensor on the hand are connected to another microcomputer that acts as the master computer, which is connected to a PC.

The PC runs the Windows 10 operating system The operating software on the master and slave microcomputers (Arduino Leonardo) was developed in the Arduino language. The control program was developed in Windows 10 and installed on the microcomputers. The microcomputers receive values from the sensors and send signals to the master microcomputer. The motors on the robot are controlled by command signals from the master microcomputer.

### Strategy of retrieving a file binder from a bookshelf and an experiment

**Figure 4:**
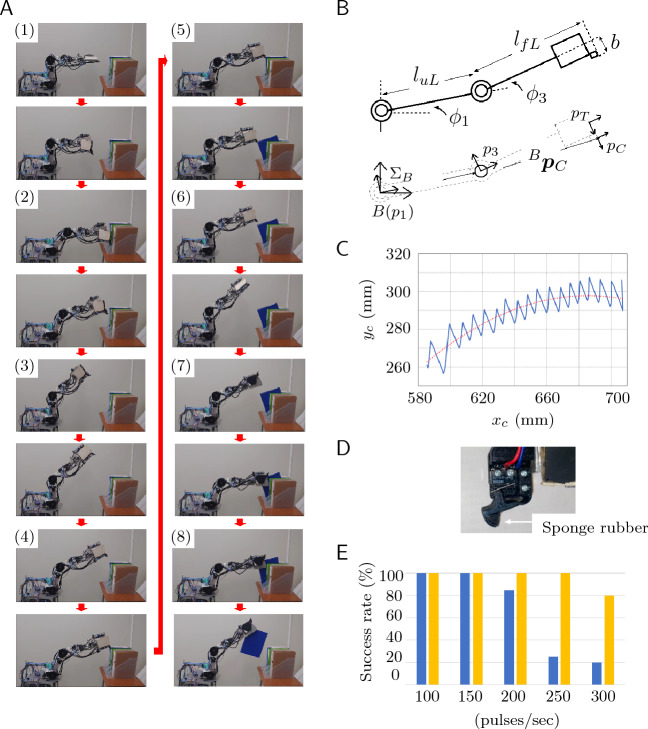
Experiment. (**A**) Process steps for retrieving the file binder. (**B**) Model showing the manipulator. (**C**) Trajectory when the binder is tilted. Solid line: calculated value using Eq. (2) and the measured value; dotted line: graph of polynomial approximation of (**B**) and (**D**). Sponge rubber glued to the claw mechanism. (**E**) Relationship between the rotational speed of the shoulder joint and the success rate. Blue bars: success rates in the case where nothing is glued to the claw mechanism; yellow bars: success rates in the case that a rubber sponge is glued to the claw mechanism.

Figure 4A shows the experiment of file binder retrieval using the robot. The retrieval process is divided into the 8 steps described below (Fig. 4A). Here, (1) to (8) correspond to the steps depicted in Fig. 4A. The authors assumed that the robot knows the height of the file binder.

The robot begins to move automatically through the process described below after receiving a start command.

1. The robot folds the left and right fingers of the hand as $\phi _7$=-90 deg, $\phi _8$=-180 deg, $\phi _9$=90 deg, and $\phi _{10}$=-180 deg (Fig. 1E).
2. The robot opens the space between the two fingers, $\phi _6$, and the ultrasonic sensor detects the distance from the hand to the file binder. The robot moves its hand forward to the position at which the robot can engage with the binder. After that, the robot stops and raises the robotic hand above the binder.
3. The robot continues moving the robotic hand upward. When the shoulder joint (Fig. 1B), $J_1$, reaches 90 deg, the robot stops moving the hand upward. The robot closes the space between the two fingers.
4. The robot lowers the hand using the shoulder joint. The elbow joint retains its angle, $\phi _3$, while the hand lowers. The switch sensor on the claw mechanism (Fig. 1F) detects contact with the top of the binder.
5. The shoulder joint (upper arm motion) continues driving to pull the top of the file binder toward the robot. The elbow joint (forearm motion) is immobile while the claw mechanism detects contact with the binder. When the claw mechanism detects contact, the hand is removed from the binder, and the robot lowers the forearm link until the claw mechanism contacts the file again.
6. The robot repeats these actions until the binder is tilted. As a result, the hand tilts the file binder by tracing a pseudo-curve path. When the robot detects the target inclination of the binder by the hand position, the robot stops the pulling the binder. The robot stops and raises the robotic hand above the binder. The robot then folds the left and right fingers to pinch the object as shown in Fig. 6G ($\phi _7=90$ deg, $\phi _8=-180$ deg, $\phi _9=90$ deg, $\phi _{10}=-180$ deg,), and the robot opens the space between the two fingers.
7. The robot lowers the hand to the height at which the file binder can be pinched. The left and right fingers are closed to grasp the binder, which is detected by the film sensors set on the fingers.
8. The robot grasps and lifts the binder by driving the shoulder axis, and the process of taking the file binder from the bookshelf is completed.

The size of file binder used by the authors in the experiment was 315 mm high, 246 mm wide, and 42 mm thick, and the mass was 260 g. The binder material was polypropylene, and the friction coefficient between the binder and the hand was $\mu = 0.68$. The shoulder joint (upper arm motion) continues driving to pull the binder in the process of tilting it (see Section "Strategy of retrieving a file binder from a bookshelf and an experiment"), and the first experiment was conducted under the conditions that the shoulder joint was rotating at a maximum of 100 pulses/min from the microcomputer. The elbow joint was driven by setting the pulse value to a maximum of 1233 pulses/min until the claw mechanism detects contact with the binder. However, the switch sensor on the claw mechanism needs to be turned on and off repeatedly over a short period of time to tilt the binder. Therefore, the actual maximum (measured) value of the elbow joint while tilting the file was 147 pulses/sec.

The shoulder and elbow joints of the robot each rotate 0.025 deg per pulse. When the pulse value from the microcomputer is *P* pulses/sec, the revolutions per minute at the shoulder joint, $N_s$, or the elbow joint, $N_e$, are shown as described below.

1 $$\begin{aligned} N_{s, e} = \frac{P \times 0.025 \times 60}{360} \hspace{1cm}\mathsf{rpm/min}. \end{aligned}$$

Thus, the experiment was conducted with a shoulder joint speed of 0.417 rpm/min, and the maximum elbow joint speed was 0.6125 rpm/min.

The experiment clarified that it is possible to retrieve the binder after performing the tilting operation. However, there were also cases of failure. Details of these and improvement methods will be described in Sections "Relationship between rotation speed of the shoulder joint and success rate of tilting the binder" and "Recovery control in case of binder tilting operation failure".

### Trajectory of claw mechanism in tilting the binder

The trajectory of the claw mechanism is generated using the measured values of shoulder and elbow joint angles. Fig. 4B is a model showing the robot manipulator. The basic robot coordinate system is denoted by $\Sigma _{B}$, where point $B (p_1)$ is the origin, $J_1$ is the shoulder axis, $p_3$ is the elbow axis, $p_t$ is the location of the tip of the hand, $p_{C}$ is the location of the claw mechanism, $l_{uL}$ is the length of the upper arm link, $l_{fL}$ is the length of the forearm link, *b* is the distance from $p_t$ to $p_{C}$, and $\phi _1$ and $\phi _3$ are the respective angles of the upper arm and forearm links. The position vectors for these joints in system $\Sigma _B$ are expressed as ${^B \varvec{p}_{i}} =[x_{i} \ \ y_i]^T$ ($i=1, 3, t, C$). It can be seen that when $\Sigma _{i}$ is parallel to $\Sigma _B$ in the local coordinate system, ${^B\varvec{p}_{1}}=[0 \ \ 0]^T$, ${^{1} \varvec{p}_{3}}=[l_{uL} \ \ 0]^T$, ${^{3} \varvec{p}_{t}}=[l_{fL} \ \ 0]^T$, and ${^{t} \varvec{p}_{C}}=[0\ \ -b]^T$. The location of the claw mechanism, $^B \varvec{p}_{C}$, is described as

2 $$\begin{aligned} {}^B \varvec{p}_{C}: \left[ \begin{array}{c} x_{C} \\ y_{C} \end{array} \right] = \left[ \begin{array}{cc} \cos \phi _{13} &{} -\sin \phi _{13} \\ \sin \phi _{13} &{} \ \ \cos \phi _{13} \end{array} \right] \left[ \begin{array}{c} l_C\\ -b \end{array} \right] + l_{uL}\left[ \begin{array}{c} \cos \phi _1 \\ \sin \phi _1 \end{array} \right] , \end{aligned}$$

where $$\phi _{13}=\phi _1+\phi _3$$.

The solid curve in Fig. 4C shows the trajectory of the claw mechanism during the tilting process of the binder, which is generated by substituting the measured values of $\phi _1$ and $\phi _3$ into (2). Here, the horizontal axis of Fig. 4C shows the distance from the shoulder axis, $J_1$, and the vertical axis shows the height from the shoulder axis. In the process of tilting the binder, the forearm link continues increasing its angle, $\phi _1$. Moreover, the forearm link repeats downward motions and stops. Therefore, the trajectory of the claw mechanism descends curvilinearly while changing the waveform.

A polynomial approximation of the forearm trajectory is shown by the dotted curve, which was generated using the polynomial approximation function of Microsoft Excel 2021 (the degree is 2). The curve shows that the trajectory of the claw mechanism rises somewhat once, but the overall direction is gently downward. This shows that it is possible to generate a pseudo-curved trajectory using the switch sensor, and the robotic hand has a practical ability to tilt the file binder.

### Relationship between rotation speed of the shoulder joint and success rate of tilting the binder

When the robotic hand tilted the binder, it was confirmed that as the rotational speed of upper arm link increased, the claw mechanism became dislodged from the binder more often. Therefore, the rotational speed of the shoulder joint and the success rate of the binder tilting operation were measured. The blue bars in Fig. 4E show the success rate to retrieve the binder when the shoulder angle was rotated by 100, 150, 200, 250, and 300 pulses/sec (0.42, 0.63, 0.83, 1.04, and 1.25 rpm/min, respectively). Here, the horizontal axis shows the rotational speed and the vertical axis shows the success rate. The experiment was repeated 20 times at each shoulder joint rotation speed, and “success” was achieved when the robot retrieved the binder. The success rates declined at a rotation of around 200 pulses/sec and significantly declined at around 250 pulses/sec.

The authors surmised that the reason for the claw dislodging from the tilting binder was one or both of the two points described below:

1. The speed of the downward rotating (clockwise relative to the paper in Fig. 4A) forearm link cannot keep up with the motion of the upward rotating (counterclockwise relative to the paper in Fig. 4A) upper arm link, causing dislodgement.
2. When the rotation speed of the shoulder joint increases, the stopping and lowering of the elbow joint repeat rapidly, which vibrates the claw mechanism link and disengages it from the binder.

Therefore, the authors prepared a polyurethane rubber sponge (Misumi Corp., SGNS5) to make it 5 mm thick, and it was affixed to the tip of the claw mechanism to add flexibility (Fig. 1D), and additional experiments were conducted. The yellow bars in Fig. 1E show the success rates to tilt the binder using the claw mechanism modified with sponge rubber.

The results of additional experiments showed that the success rate increased significantly. To realize a stable binder tilt using the proposed system, it was clarified that slightly flexible hardware is needed; moreover, improving the software to handle the case is needed when the tilting maneuver fails.

### Recovery control in case of binder tilting operation failure

Based on the results described above, a control system that can recover the operation when the robot fails to retrieve the binder was constructed. When the claw mechanism detaches from the binder, the forearm link continues to rotate downward (clockwise relative to the paper in Fig. 4A). Therefore, the control system in which the robot identifies failure to tilt a binder using the amount of change of the forearm link angle, $\phi _3$, was constructed to perform a recovery operation.

Equation (3) shows the amount of change of the forearm link angle per unit time. Here, $\Delta t$ is the sampling time, $\Delta \phi _3$ is the amount of change of the elbow joint angle, and *D* is a set value.

3 $$\begin{aligned} D \le \frac{\phi _3}{\Delta t}. \end{aligned}$$

**Figure 5:**
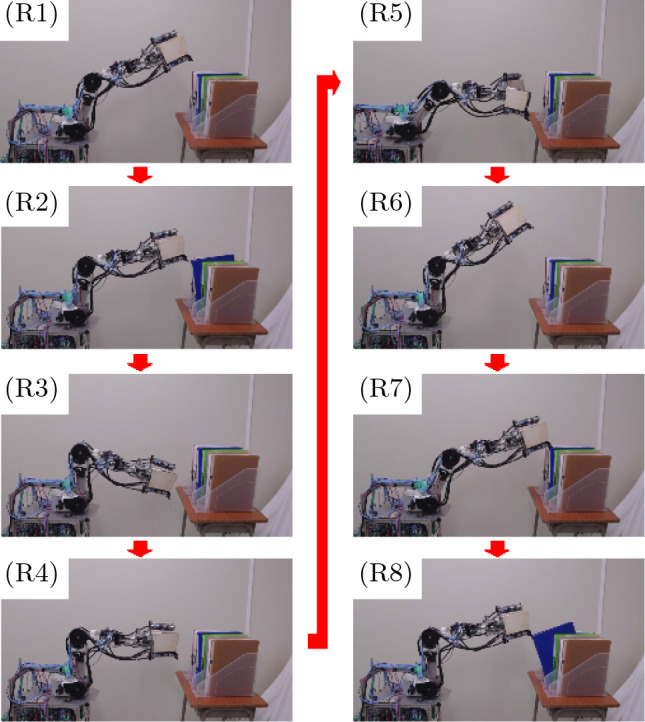
Experiment of recovery when the robotic hand comes loose from the binder and fails to tilt it.

When (3) is satisfied, the robot recognizes dislodgement from the binder and begins a recovery operation. When the robot tilt the binder with a maximum shoulder joint velocity of 100 pulses/sec, the maximum velocity of the elbow joint was 147 pulses/sec (measured value, see Section "Strategy of retrieving a file binder from a bookshelf and an experiment"). Therefore, the value of *D* should be set to over 147 pulses/sec. The authors constructed a control program such that, when the elbow joint rotates downward at over 300 pulses/sec, the robot recognizes this as tilt motion failure and reattempts to retrieve the binder. [Because the forearm link is driven by 0.025 deg/pulse (see Section "Strategy of retrieving a file binder from a bookshelf and an experiment"), $D=7.5$ deg/sec when the forearm link is driven at 300 pulses/sec.] Figure 5 shows the recovery motion experiment, which validated the effectiveness of the control system.

The retrieval recovery tilting process is described below, where (R1) to (R8) correspond to the steps depicted in Fig. 5.

1. The robot lowers the hand using the shoulder joint.
2. The upper arm link continues rotating toward the top. The forearm link is immobile while the claw mechanism detects contact with the binder. When the claw mechanism is found to have lost contact with the binder, the robot lowers the forearm link until the claw mechanism contacts the file again.
3. Once the claw mechanism is completely dislodged from the file binder, the forearm link continues to rotate downward.
4. When the operation in (R3) is complete, the robot recognizes dislodgement from the binder and begins a recovery operation.
5. The ultrasonic sensor detects the distance from the hand to the file binder.
6. The robot moves the robotic hand upward.
7. The robot lowers the hand using the shoulder joint until the claw mechanism detects contact with the file binder .
8. The binder tilting process is begun again.

## Discussion

This paper describes the details of a foldable robotic hand and a control method to retrieve file binders placed on a bookshelf. Specifically, a control method to retrieve a binder by using inexpensive switch sensors is proposed and its effectiveness is confirmed through experiments. The details are summarized below.

1. The robotic hand was developed by focusing on the way the humans handle such objects, which revealed that only 6 DOFs are needed to handle various objects. The claw mechanism to snag an object was made using a 3D printer and it was attached to the side of the robotic hand. The claw mechanism has a simple structure, with only one microswitch sensor. In the process to retrieve a binder, the robot has to tilt it and then use the claw mechanism to generate a pseudo-curved trajectory in the tilting process. Experimental results demonstrated the effectiveness of the proposed method.
2. Next, experiments to clarify the success rate of the operation were conducted. The results showed that the success rates of retrieving a binder are largely influenced by the speed of the upper arm link. The authors concluded that the reasons for the tilt failure were twofold, namely that the speed of the downward rotating forearm link cannot keep up with the motion of the upward rotating upper arm link and that the claw mechanism link vibrates due to the rapid and repeated contact and separation between the binder and the robotic hand. Therefore, the hand was improved by the addition of a rubber sponge to provide a compliance function in the claw mechanism. Re-evaluation of the success rate validated a significant improvement.
3. In addition, a recovery control when the robot fails to tilt the binder was also developed. The judgment of failure of binder tilting is based on the amount of change in the forearm link angle, and the system was able to recognize this without the need for another sensor. The experimental results showed the effectiveness of the recovery control.

In this study, the space above the bookshelf was assumed to be empty, so there is a need to investigate other various manipulation link speeds and various situations of object placement.

In addition, depending on the size of the file and the environment in which the file is located, it may not be possible to retrieve the file using the proposed method. For example, the coefficient of friction between the file and the bookshelf may be lower than expected, or the direction of the force when the hand is lowered to the file may shift the position of the file significantly. In order to adapt the system to such cases, it is necessary to conduct an exact theoretical analysis and experiments that take into account various factors when executing file retrieval and, based on the results, build a control system with a high success rate while maintaining low-cost feasibility. It is also necessary to verify the effectiveness of this method by conducting similar experiments using robots equipped with other manipulators. In this case, the authors are considering using the encoder values provided by the fingers of a multi-fingered hand instead of the touch sensor-based claw mechanism reported in this paper. Thus, there are still many improvements to be made. However, it is noteworthy that our method demonstrated that a simple system using inexpensive sensors can generate a curved trajectory along the inclination of an object and perform object retrieval. In addition, the proposed method can be adapted to the orbital motion of the rotating mechanisms that exist in many human living spaces, and it can be used for not only the robot the authors developed but also many other human-assistive robots. Our approach has the potential to revolutionize the sensor configuration required for human-assistive robots and significantly reduce the required cost.

In the future, the authors plan to conduct experiments for grasping objects of various materials and masses, such as files, books, and notebooks, to elucidate the optimal conditions for the compliance function and recovery control. A theoretical analysis is performed taking into account the various file types, their placement, and the misalignment caused by the forces exerted by the hand. Based on the results, we will establish a method to realize ultra-precise file removal. We will also conduct similar experiments using another mobile robot to verify the effectiveness of this method. In addition, the authors plan to realize the retrieval of binders or books placed on a multi-tiered bookshelf, and plan to aim for higher precision in each operation to realize a control system that can generate precise curved trajectories using the method.

Furthermore, the authors also plan to aim for higher precision in each operation with the intention of examining new tactics for retrieving a file binder without using a claw mechanism.

## Methods

### Concept of the foldable robotic hand

**Figure 6:**
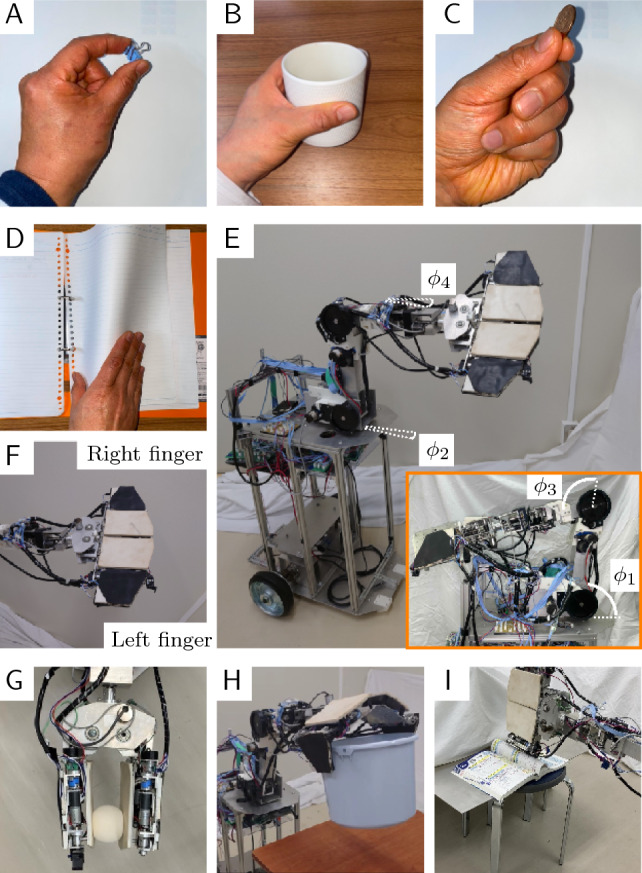
Concept of the robotic hand. (**A**) Pinching an object with a human hand. Force is exerted on one point of the object using two or three fingers. (**B**) Grasping an object. A planar force is exerted against the object. (**C**) Human fingers handling a small object. Some fingers are not participating in the object handling. (**D**) Turning pages. This is an example of a task that can be performed without continuously moving the fingers. (**E**) Overview of the robot. (**F**) Hand mechanism having planar fingers. (**G**) Pinching an object with the foldable robotic hand. (**H**) Grasping an object using the robot. (**I**) Turning pages using the robot.

A human can handle small objects by exerting a force at one or several points with the fingertips (Fig. 6A) and can grasp a fragile object by exerting a planar force with the palm (Fig. 6B). Many multi-fingered robotic hands have been developed using human or animal hands as models. Such hands require the bending and stretching of fingers to perform tasks, and a number of fingers and joints are necessary to execute advanced tasks. However, as the number of fingers increases, the mechanism becomes more complex and is more difficult to control. In other words, a robotic hand with many fingers is expensive and hard to control.

However, some tasks are performed by handling an object at specific points of the fingers, such as pinching a small object. Human fingers perform such tasks using only two or three fingers to handle the object, and some fingers do not act on the object (Fig. 6C). In addition, some tasks can be performed on an object without continuously closing or extending the fingers, such as simply lifting or placing an object. Turning pages can be also performed without continuously moving the fingers (Fig. 6D). In such cases, continuous driving of the finger axes is not needed, or any needed force is not very large. The main aim is to secure a contact area between the object and the fingers, and the fingers are used like plates. Thus, such tasks can be executed by a robotic hand with two or three plate-like fingers.

As described above, even a robotic hand that has only small number of fingers and DOFs can be expected to effectively use these fingers for many tasks by properly arranging and shaping them and increasing the movable range of joints. No multi-fingered robot hand had been developed based on the above point of view until the 6-DOF robotic hand called JINZU developed by the authors (Fig. 6E).

The robotic hand has two planar fingers with a large movable joint range (Fig. 6F). The robotic hand changes its shape, and it uses not only the front surface of the fingers but also the back surface and sides of the fingers, which humans do not frequently use. Thus, it is able to handle various objects by exerting a force at one or several points with the fingertips or a force in a plane (Fig. 6G−I).

### Size of claw mechanism

**Figure 7:**
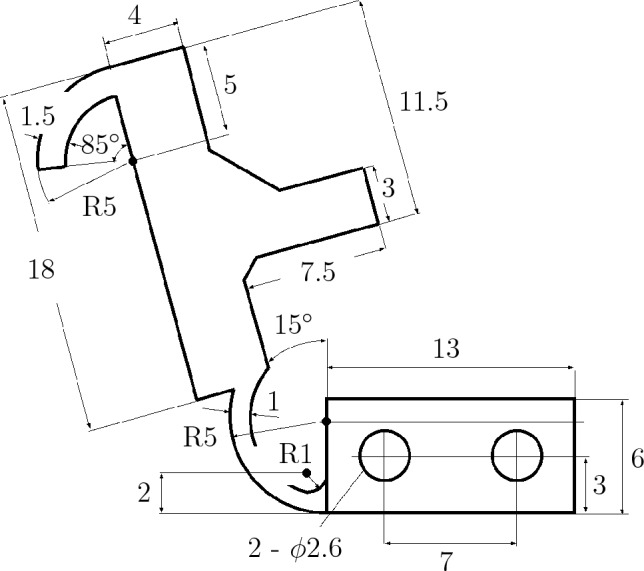
Model showing the claw mechanism and the approximate dimensions.

The claw mechanism to snag an object was made using the 3D printer as shown in Fig. 1F. A model of the claw mechanism showing the approximate dimensions is presented in Fig. 7

### Sponge rubber glued to claw mechanism

The authors used the sponge rubber SGNS 10” (Misumi Corp.) to add flexibility to the finger claw in the additional experiment (Fig. 4D). The material of the sponge is polyurethane, which has a hardness of less than 1 on the Asker hardness scale, and its apparent density is 0.022 g/cm$^3$ (information on the materials was obtained from the website of Misumi Corp.). The authors cut the sponge to make it 5 mm thick.

## Supplementary Information

Supplementary Information 1.

Supplementary Information 2.

## Data Availability

The datasets used and/or analyzed during the current study are available from the corresponding author on reasonable request.

## Code availability

The datasets used and/or analyzed during the current study are available from the corresponding author on reasonable request.
